# Probing the Effect and Mechanism of Flue Gas on the Performance of Resorcinol/Hexamethylenetetramine-Based Polymer Gel in Flue Gas Flooding Reservoir

**DOI:** 10.3390/gels8120772

**Published:** 2022-11-26

**Authors:** Wenli Qiao, Guicai Zhang, Jianda Li, Ping Jiang, Haihua Pei

**Affiliations:** School of Petroleum Engineering, China University of Petroleum (East China), Qingdao 266580, China

**Keywords:** flue gas, resorcinol/HMTA gel, gelation performance, elevated gas pressure, long-term stability

## Abstract

Polymer gel plugging is an effective method for gas mobility control in flue gas flooding reservoirs. However, the effect and mechanism of flue gas on the performance of polymer gels have rarely been reported. In this study, a polymer gel was prepared by cross-linking hydrolyzed polyacrylamide (HPAM) and resorcinol/ hexamethylenetetramine (HMTA) to illuminate the influencing mechanism of flue gas composition on gel. The gel rheological testing results showed that flue gas promoted gelation performance, whereas it seriously threatened gel long-term stability, especially at high pressure conditions. The influence of CO_2_ on the polymer gel had the characteristic of multiplicity. The hydrodynamic radius (R_h_) and the initial viscosity of HPAM solution decreased in the presence of CO_2_. Nonetheless, the dissolved CO_2_ expedited the decomposition rate of HMTA into formaldehyde, which promoted the cross-linking process of the HPAM, leading to a shorter gelation time. Oxidation–reduction potential (ORP) tests and Fourier transform infrared spectroscopy (FTIR) analysis indicated that O_2_ played a leading role in the oxidative degradation of HPAM compared to CO_2_ and threatened the gel long-term stability at elevated gas pressures. To address the adverse effects caused by flue gas, it is highly desirable to develop polymer gels by adding oxygen scavengers or strengthening additives.

## 1. Introduction

In situ combustion technology has been rapidly developed for enhanced oil recovery (EOR) in the Xinjiang oilfield in recent years. However, understanding how to properly treat the flue gas generated from combustion has become a major problem. As is known, flue gas flooding has been an environment-friendly and effective EOR method in many oilfields [1,2]. Hence, it is considered to be a promising method to improve the oil displacement efficiency in the HONG reservoir of the Xinjiang oilfield, with a low matrix permeability of 58 mD, a porosity of 17.8%, and a low reservoir temperature of 42 °C. However, flue gas tends to flow through big channels existing in reservoir, significantly reducing the sweep efficiency and oil recovery [3,4,5,6,7].

To date, polymer gel treatments have been an effective strategy for gas mobility control, including N_2_ and CO_2_ [8,9,10]. Generally, polymer gels are derived from the cross-linking process of the polymers with different types of cross-linkers [11,12]. The performance of polymer gels is a key criterion in a successful implementation of gas mobility control [13]. However, until now, there have been few reports on the performance of polymer gels in the flue gas atmosphere. To the best of our knowledge, the gel performance will be severely weakened when plugging the CO_2_ flow due to the acidic conditions created by CO_2_ [14]. Note that the flue gas generated by in situ combustion in Xinjiang oilfield is a mixture of different gases, consisting of N_2_ (80%), CO_2_ (17%), and O_2_ (3%). The gel performance during flue gas plugging would be very unpredictable. To clear the influencing mechanism of flue gas on polymer gels is of significance from both fundamental and application perspectives [15].

As is known, the performance of polymer gels is determined by many factors, including polymer properties, cross-linker behaviors, temperature, and pH [16,17]. The effect of gas types on the above factors has been extensively studied [18,19,20,21,22,23,24]. Owing to the cross-linkable hydroxymethyl groups (–CH_2_OH) and amide groups (–CONH_2_) in molecular chains, the polymer is the main agent in a polymer gel system [25]. However, the polymer chains are prone to suffer from degradation and a severe reduction in viscosity in harsh reservoir conditions [18,19]. The presence of O_2_ triggers the oxidative degradation of polymers, and substantially reduces the solution viscosity [20,21]. Gaillard et al. reported that the radicals generated by O_2_ at 85 °C could break the backbone of the polymer and lead to a reduction in the molecular weight and viscosity of the polymer solution [22]. Furthermore, the acid gas CO_2_ also impacts polymer properties. Indeed, CO_2_ dissolved in the polymer solution lowers the pH with the formation of carbonic acid, which neutralizes the negative charge on polymer chains by transforming the ionic carboxylate groups into non-ionic carboxylic acid groups, resulting in a rapid decline in viscosity [23,24]. Moreover, the long-term stabilities of polymers are also influenced by CO_2_. Tovar et al. revealed that the retention rate of the viscosity of hydrolyzed polyacrylamide (HPAM) solution in the presence of CO_2_ decreased from 54% to 30% during an aging time from 214 to 318 days at 50 °C, mainly owing to the hydrolysis of the amide groups and the induced degradation by the interaction of the divalent cations with the acrylate groups [26]. Based on the above, the influence on polymers properties induced by the flue gas, including the gelation performance as well as the gel long-term stability, is expected to be much more complicated. Cross-linkers are also crucial agents in forming polymer gels. Generally, cross-linkers are divided into two main types, as follows: inorganic cross-linkers (cross-linking with the-CH_2_OH in polymers) and organic cross-linkers (cross-linking with the –CONH_2_ in polymers). Inorganic cross-linkers mainly contain Al^3+^, Zr^4+^, Cr^3+^, etc., [25,27]. In recent years, chromium acetate has been widely employed to form gels due to its lower toxicity and longer gelation time [28,29]. However, previous studies reported that the cross-linker behaviors are strongly affected by acid gas. According to Unomah et al., the decrease in pH originating from the dissolved acid gas led to a poor gelation performance due to the depletion of tCr^3+^ [30]. Moreover, the pH is also a key chemical parameter in the structure of chromium acetate. Decreasing the pH value would retard the decomposition rate of the chromium acetate cyclic trimer into a linear trimer, conceivably leading to a reduction in cross-linking density and a weak gel strength [31]. As for organic cross-linkers, they mainly consist of aldehydes, phenol/aldehyde, phenolic resins, polyethyleneimine (PEI), and so forth [32,33,34]. Among them, phenolic/aldehyde is broadly utilized to form gels. Moreover, resorcinol and hydroquinone are more widely adopted as substitutes for the toxic phenol, and HMTA is a less toxic substitute for aldehyde [35]. A valuable advantage of the phenolic/aldehyde cross-linked gel is the longer gelation time compared to chromium-cross-linked gels, which provides sufficient time for the gelant to penetrate into the deep formation in low-permeability reservoirs [36]. Several studies have reported that the behavior of organic cross-linkers is affected by acidic materials [37]. For instance, acids stimulate the decomposition of HMTA into formaldehyde, facilitating the cross-linking process from gelant to bulk gel [38]. Resorcinol and hydroquinone suffer from oxidation side reaction due to O_2_, probably leading to a reduction in cross-linking sites and a poor gel strength [39]. Research results reveal that the acid gas and O_2_ contained in flue gas would have a more intricate effect on cross-linker behaviors.

Considering the influence of gases on polymer properties and cross-linker behaviors, we can image that the performance of polymer gels in the presence of flue gas will be more complex, especially for high gas injection pressure conditions in reservoirs. Herein, the objective of this work was to probe the influence of flue gas on the performance of polymer gels and to elucidate the effect mechanism. The polymer gel system was prepared by cross-linking HPAM and resorcinol/HMTA cross-linker. Storage modulus measurements were adopted to characterize the gelation behavior and gel performance. Analytical testing methods including dynamic light scattering (DLS) tests, rheological experiments, oxidation–reduction potential (OPR) measurements, and Fourier transform infrared spectroscopy (FTIR) analyses were adopted to comprehensively investigate the effect mechanism of the flue gas. This work aids in the development of an advanced polymer gel formulation that is suitable for flue gas flooding reservoirs.

## 2. Results and Discussion

### 2.1. Preparation of Gel Formulation

**Figure 1:**
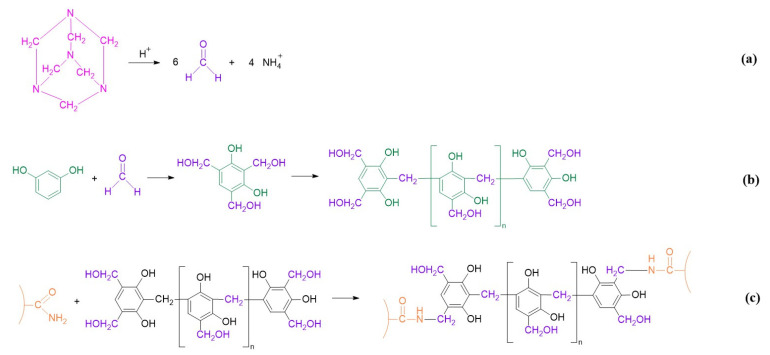
Reaction process of resorcinol /HMTA-based polymer gel. (**a**) Decomposition of HMTA into formaldehyde; (**b**) the generation of oligomers of phenolic resin; (**c**) cross-linking process between the phenolic resin and the HPAM.

The resorcinol/HMTA-based polymer gel system is formed by cross-linking HPAM with the product of resorcinol and HMTA. The reaction processes are illustrated in Figure 1. Firstly, HMTA was gradually decomposed into formaldehyde with the aid of an acidic catalyst (Figure 1a). Secondly, formaldehyde reacted with the phenolic hydroxyl groups originating from resorcinol and formed into oligomers of phenolic resin (Figure 1b). Subsequently, the –CH_2_OH in the phenolic resin cross-linked with the –CONH_2_ in HPAM to form a bulk gel (Figure 1c) [40].

**Figure 2:**
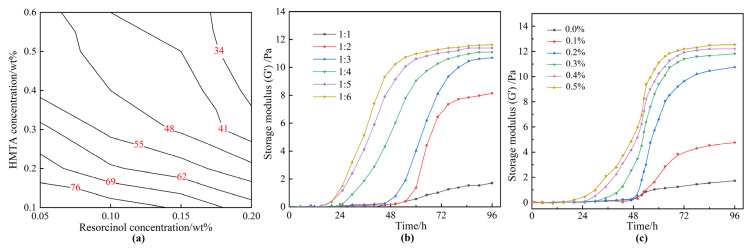
The gelation performance of resorcinol/HMTA-based polymer gel. (**a**) Effect of the concentrations of resorcinol and HMTA on the gelation time; (**b**) relationship between the storage modulus (G′) of gels and the aging time as well as the mass ratio of resorcinol to HMTA; (**c**) effect of the concentrations of acetic acid on the gelation time.

**Figure 3:**
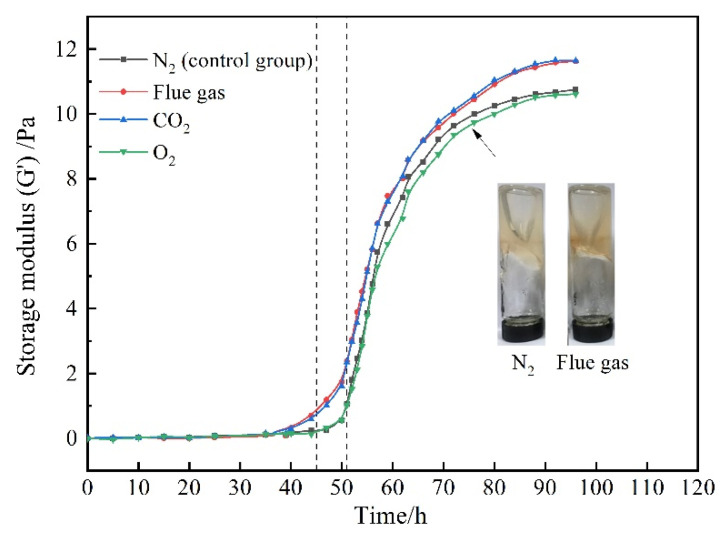
The influence of gas species on gelation behavior.

As is known, the gelation time and the gel strength are key indicators for gas mobility control. The contour map revealing the effect of the concentrations of resorcinol (0.05–0.2 wt%) and HMTA (0.1–0.6 wt%) on the gelation time is represented in Figure 2a. According to Sydansk and Southwell, low molecular weight polymers (<2 million) are typically employed to treat <200 mD permeability reservoirs [41]. In our previous work, only a few commercial polymers with a molecular weight of less than 2 million were available, and a mass of polymer was required to form a strong bulk gel, resulting in higher costs. Hence, a commercial HPAM with a molecular weight of 2 million was optimized to ensure an excellent injectivity and a strong strength polymer gel. Moreover, the concentration of HPAM was fixed at 0.5 wt%. To catalyze the gelation reaction, 0.2 wt% acetic acid was introduced into the gelant. Acetic acid is a weak acid compared to hydrochloric acid and relieves the damage to reservoirs. According to Figure 2a, increasing the concentration of resorcinol or HMTA shortened the gelation time. Noteworthily, HMTA played a much more important role in promoting the gelation process. Therefore, to further explain the influence of HMTA on gel strength, the relationship of the storage modulus (G′) of gels with the aging time, as well as the dosage of HMTA, was explored. The concentration of resorcinol was fixed at 0.1 wt%. The mass ratio of resorcinol to HMTA varied from 1:1 to 1:6. As displayed in Figure 2b, when the mass ratio of resorcinol to HMTA was 1:1 or 1:2, a weak gel was formed with a much slower gelation rate. As the mass ratio of resorcinol to HMTA increased to 1:3 or more, the gelation time was shortened, and a bulk gel was formed within 60 h, with a G′ of more than 10 Pa. Previous studies suggested that the reasonable mass ratio of phenol to HMTA is 1:1 in high-temperature reservoirs [38]. However, for the low temperature of 42 °C, HMTA needs to be superfluous in order to decompose into sufficient formaldehyde, which facilitates the following cross-linking process. Moreover, the gelation rate is highly dependent on the concentration of acetic acid, as exhibited in Figure 3c. The gelant could not form into gels within 96 h by employing less than 0.1 wt% acetic acid, as only a small quantity of formaldehyde was generated from the decomposition of HMTA. When the addition of acetic acid increased to 0.2 wt%, the gelant was able to form into bulk gels within 72 h. Meanwhile, the gelation time was shortened at elevated acetic acid concentration, owing to the accelerated decomposition rate of the HMTA.

### 2.2. Effect of Flue Gas on Gelation Behavior and Gel Performance

After displacing the gelant into target regions, the wells should be shut in for several days until the gelant forms into gels. However, the residual flue gas in the previous gas injection process is still present in the formation. Thus, the chemical condition will be altered due to the unavoidable penetration of the flue gas into the polymer gelant. Therefore, the cross-linking process of HPAM might be incomplete. Hence, the influence of flue gas on gelation time, gel strength, and gel long-term stability should be taken into consideration.

The gelants (consisting of 0.5 wt% HPAM, 0.3 wt% HMTA, 0.1 wt% resorcinol, and 0.2 wt% acetic acid) were prepared through saturation with different gases. The variation of G′ with gas species and aging time is presented in Figure 3. In view of the chemical inertness of N_2_, the gelant treated with N_2_ was adopted as the control group. In terms of the control group, the G′ of the gelant barely changed at the initial stage and then increased rapidly. The gelation time was about 51 h, and the final G′ of the polymer gel reached up to 10 Pa after being aged for 72 h. Notably, as the gelant was saturated with flue gas, the increasing rate of the gel strength sped up. The gelation time was shortened to about 45 h. Meanwhile, the gel strength was much higher compared to that of the control group. The final G′ of the polymer gel increased to 12 Pa. The flue gas positively promoted the gelation performance, as it compensates for the disadvantage of the postponed gelation process and reduced gel strength caused by the inevitable gravity differentiation and chromatographic separation in the process of gelant migration in the rock matrix.

To further specify the influence of the composition of flue gas on the gelation behavior, the gelants treated with composited O_2_ and composited CO_2_ were further explored. For convenience, the composited O_2_ and composited CO_2_ are abbreviated as O_2_ and CO_2_ in the following text. As revealed in Figure 3, O_2_ barely changed the gelation behavior compared to the control group, while CO_2_ notably shortened the gelation time to 45 h and increased promotes the G′ of the polymer gel to 12 Pa, indicating that the CO_2_ in flue gas positively promoted the gelation process and gel performance.

**Figure 4:**
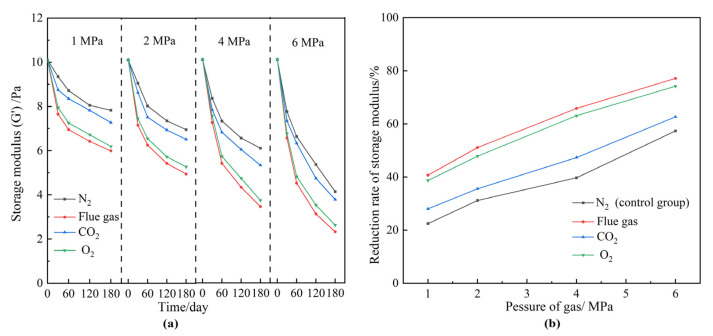
The effect of gas species and gas pressures on gel long-term stability. (**a**) G′ of gels at elevated gas pressures versus aging time; (**b**) reduction rate of gel strength after aging for 180 days.

**Figure 5:**
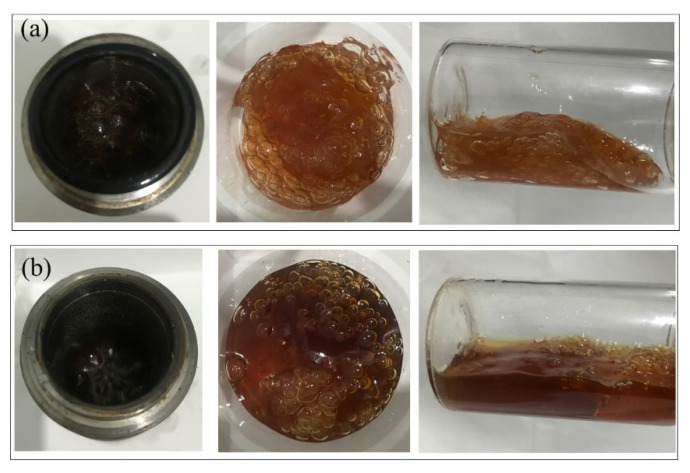
Gel morphology after aging for 180 days at 2 MPa. (**a**) Control group—N_2_; (**b**) flue gas.

Ideally, large pores and fractures should be plugged by polymer gels during the post-flue gas treatment. However, the gas injection pressure reaches up to several megapascals. As a matter of fact, the squeeze and penetration of the high-pressure flue gas may weaken the gel strength. Moreover, the required validity period (more than 180 days) for polymer gel treatment means that the long-term stability of the polymer gel is vital to realizing practical applications. A series of polymer gels prepared under the same conditions were aged in the presence of different gas types and pressures. The gas types included N_2_, flue gas, O_2_, and CO_2_. The gas pressure ranged from 1 to 6 MPa. The aging time was 180 days. Figure 4 reveals the effect of gas types and gas pressures on the gel long-term stability during the aging process. The gels treated with N_2_ were adopted as control groups. According to Figure 4a, the gel strengths decreased with the aging time and gas pressure. The relationships between the reduction rates of gel strengths and the gas pressures are summarized in Figure 4b. The long-term stability of gels suffered more severe damage at elevated pressures. The reduction rate of gel strength increased from 20% to 80% as the gas pressure increased from 1 MPa to 6 MPa. Moreover, the overall reduction rates of gel strengths in flue gas, O_2_, and CO_2_ were much higher than that of N_2_. Given the chemical inertness of N_2_, the reduction in gel strength in the control group resulted from the physical damage caused by the extrusion of the high-pressure gas. In addition to physical damage, high-pressure CO_2_ and O_2_ may also affect the cross-linking and stabilization processes of HPAM, which in turn leads to a further reduction in the strength of the polymer gel, as will be confirmed in subsequent chapters. Take 2 MPa, for example; the G′ of the polymer gel treated with N_2_ decreased by 31.2% after 180 days, mainly resulting from the physical destruction by the high gas pressure. This value decreased to 51.1%, 47.8%, and 35.5% in the flue gas, O_2_, and CO_2_ groups, respectively. The gel in the control group still held together and maintained a medium-high strength after 180 days at 2 MPa. By contrast, the gel was significantly destroyed in flue gas and became more fragile (Figure 5). Collectively, flue gas seriously damaged the gel’s long-term stability at elevated gas pressures. In particular, the impact of O_2_ in flue gas was more pronounced than that of CO_2_.

Overall, the flue gas promoted the gelation process of resorcinol/HMTA gels while damaging the gel long-term stability, inevitably resulting in increasing failure in gas mobility control. Further studies were carried out to illuminate the effect mechanism of the flue gas and its composition on gelation behavior and gel performance.

### 2.3. Effect Mechanism on Gelation Performance

#### 2.3.1. HPAM Properties

**Figure 6:**
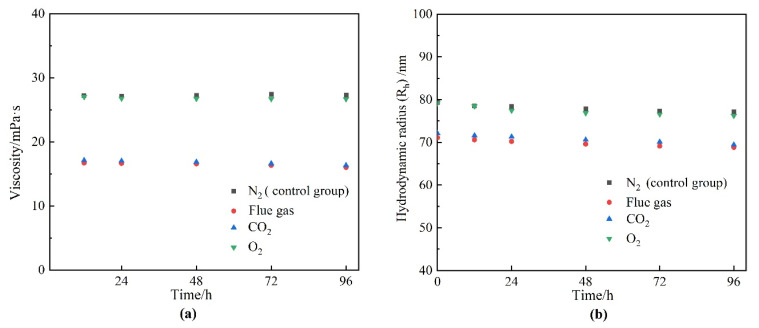
The effect of gas types on HPAM properties. (**a**) Viscosity; (**b**) hydrodynamic radius (R_h_).

**Figure 7:**
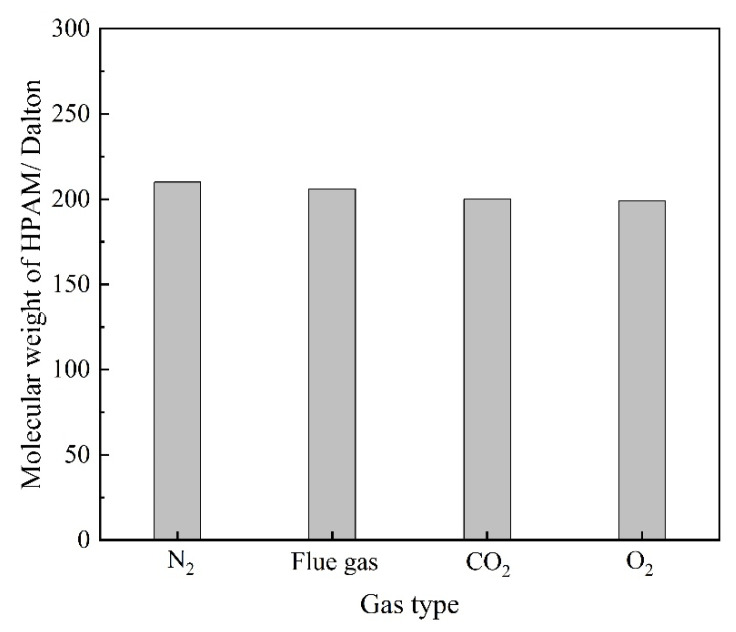
The changes in the molecular weight of HPAM treatment with different gas.

Components in the flue gas may influence the underlying properties of HPAM and, thus, the gelation behavior. The influence of gas types on the viscosity as well as the hydrodynamic radius (R_h_) of HPAM solution is illustrated in Figure 6. The HPAM solutions were saturated with different types of gas under atmospheric pressure and then aged for 12–96 h. The viscosity of the HPAM solution in the control group (saturated with N_2_) remained almost constant during the aging process. The viscosity of the O_2_ group did not decrease with aging time as expected, indicating that the oxidative degradation of HPAM does not take place during the aging time of several days at low temperature. Instead, upon contact with the flue gas as well as CO_2_, the viscosities of the HPAM solutions were likewise reduced by 37% compared to the control group. The reduction in solution viscosity might be produced by the degradation of HPAM and changes in the configuration alteration of the molecular chains. The changes in the molecular weight of HPAM before and after treatment are compared in Figure 7. It can be observed that the flue gas and CO_2_ had little effect on the viscosity-average molecular weight of HPAM, indicating that the reduction in solution viscosity resulted from the configuration alteration of HPAM molecules. The conformational transition of the molecular chain causes a change in the R_h_ of HPAM, which can be indirectly characterized by dynamic light scattering (DLS) measurement. Hence, the variation in the R_h_ of HPAM before and after treatment was studied, as shown in Figure 6b. As expected, the R_h_ of HPAM followed the same trend as the solution viscosity. Meanwhile, it is shown that the pH was reduced from 8.78 to 6.86 when the HPAM solution was treated with flue gas and CO_2_. The decrease in the pH of the HPAM solution was due to the dissolution of CO_2_, thus, promoting the conversion of the sodium carboxylate group (–COONa) to carboxyl groups (–COOH). The reduction of carboxylate groups (–COO^−^) induced the weakness of electrostatic repulsion, turning the stretched polymer chains into curls, resulting in a sharp reduction in R_h_ and viscosity [24].

The reduction in R_h_ of HPAM reduced the collision rate between the cross-linker and the polymer, thus, reducing the cross-linking density in the polymer gel, which threatened the gel strength. However, according to Section 2.2, the presence of CO_2_ in flue gas positively promoted the gelation process and gel performance. Hence, it was necessary to further investigate the effect of the presence of CO_2_ on the cross-linker and the cross-linking process.

#### 2.3.2. Cross-Linker Behavior

**Figure 8:**
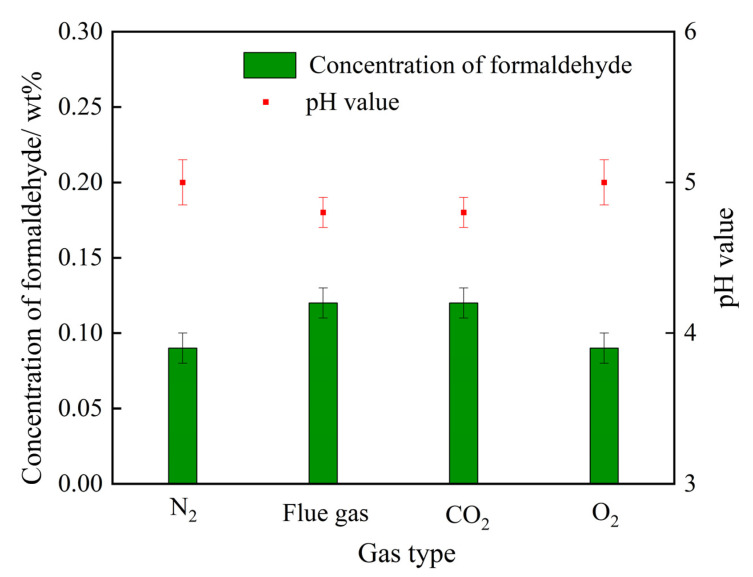
The effect of gas type on the formaldehyde concentration and the pH value of the HMTA solution.

As illustrated in Figure 1, the step of the decomposition of HMTA into formaldehyde plays a dominating role in the generation rate of cross-linkers. A higher concentration of formaldehyde not only means a higher decomposition rate of HMTA, but also many more cross-linking sites and, thus, a faster cross-linking reaction rate. The variation trends of formaldehyde concentration in the HMTA solutions are explored afterwards. A series of 0.3 wt% HMTA solutions were prepared through treatment with N_2_ (control group), flue gas, CO_2_, and O_2_, separately. As depicted in Figure 8, O_2_ had little influence on the concentration of formaldehyde. By contrast, the concentrations of formaldehyde in the HMTA solutions were significantly elevated after being treated with both flue gas and CO_2_, signifying that CO_2_ in the flue gas may dominate in the decomposition rate of HMTA to formaldehyde. Compared to the HMTA solution in the control group, the pH value decreased from 5.1 to 4.8 during the flue gas and CO_2_ treatment. Hence, the reduction in pH was the main reason for the expedited decomposition rate of HMTA.

**Figure 9:**
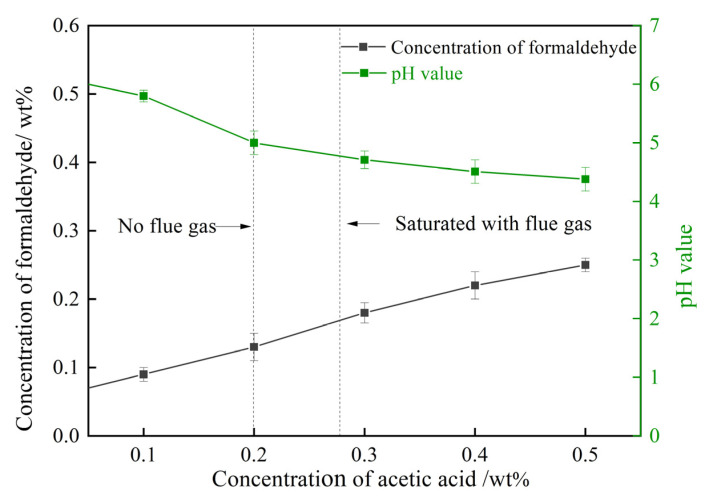
Effect of acetic acid on the decomposition of HMTA.

Acetic acid mainly acts as a pH-adjusting agent in gelant, triggering the decomposition of HMTA. Thus, 0.3 wt% HMTA solutions with 0.1–0.5 wt% acetic acid were prepared. The changes in pH and the concentration of formaldehyde triggered by acetic acid are studied in Figure 9. The pH value decreased from 6.2 to 4.38 as the concentration of acetic acid in the HMTA solution increased from 0 to 0.5 wt%. The concentration of formaldehyde, thus, rose from 0.07 wt% to 0.22 wt% due to the enhanced acid catalyst. As for the HMTA solution treated with flue gas, the dissolution of CO_2_ in the solution paralleled the increase in the concentration of acetic acid. The dissolved CO_2_ reduced the pH value of the gelant and expedited the decomposition rate of HMTA, thus, promoting the production of formaldehyde, which contributed to the cross-linking process.

**Figure 10:**
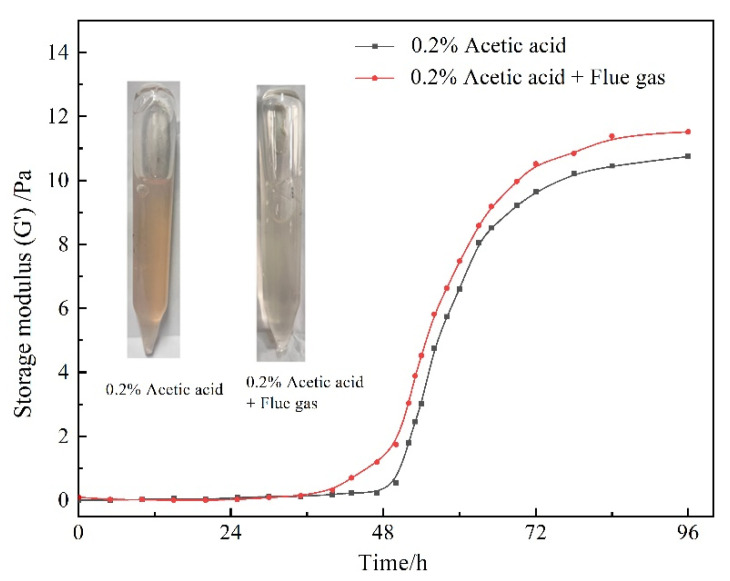
Gelation performance of gelant with 0.2 wt% acetic acid and flue gas.

As a cross-linking agent, resorcinol is susceptible to oxidation to form benzoquinone, characterized by the color of the solution turning from colorless to pink. The O_2_ in flue gas exacerbates this reaction rate. Meanwhile, the oxidation reaction is also influenced by the pH of the gelant. Our previous experiment indicated that the acid environment originating from acetic acid alleviated the oxidation reaction of resorcinol (images in Figure 10). From this perspective, the CO_2_ in flue gas led to a desirable decrease in the pH value and contributed to the resistance of the oxidation reaction. Combining the oxidation resistance effect with the gelation performance in Figure 10, it can be concluded that the flue gas expedites the decomposition of HMTA and the generation of cross-linkers, promoting the cross-linking process of HPAM.

### 2.4. Effect Mechanism on Long-Term Stability

#### 2.4.1. Long-Term Stability of HPAM

**Figure 11:**
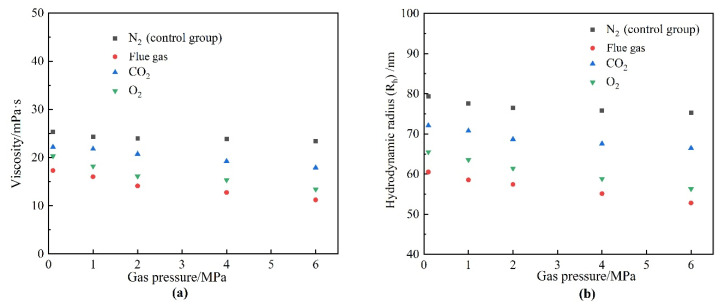
The properties of HPAM after aging for 180 days in different gases as a function of gas pressure. (**a**) Viscosity; (**b**) hydrodynamic radius (R_h_).

**Figure 12:**
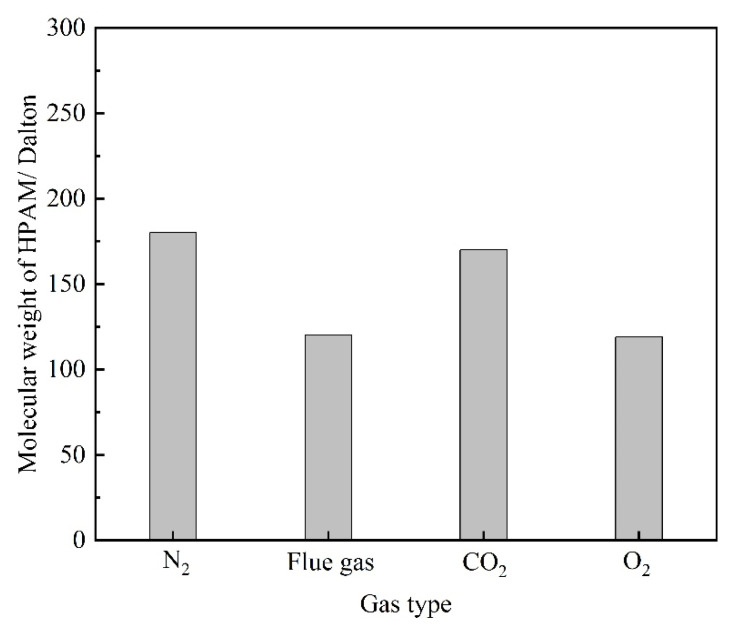
The changes in the molecular weight of HPAM treatment with different gas.

Flue gas seriously damages the gel long-term stability at elevated gas pressures and inevitably results in an increasing failure in gas mobility control. The reduction in the strength of the polymer gels might result from two reasons. On the one hand, high-pressure flue gas may impose physical damage on the polymer gel through extrusion and penetration, causing the rupture of the polymer gel, which is verified by the control group in Figure 4a. On the other hand, the dissolution of CO_2_ and O_2_ alters the chemical environment of the formation fluid and may inhibit the cross-linking process or disrupt the chemical structure of the polymer gel, which is also the key part of this subsection. To explore the stability performance of polymers, the viscosity and R_h_ of HPAM solutions aged for 180 days at elevated gas pressures were evaluated. The effect of gas type and gas pressure on the viscosity of HPAM is illustrated in Figure 11. Similarly, the HPAM solution treated with N_2_ was once again adopted as the control group. As revealed by Figure 11a, the increase in N_2_ pressure had little influence on the viscosity of the HPAM solution and almost stayed constant at 25 mPa·s. On the contrary, a rapid decline in viscosity occurred at elevated flue gas pressures. Meanwhile, both CO_2_ and O_2_ in flue gas inflicted serious damaged on HPAM viscosity at elevated gas pressures. Although the viscosity and R_h_ of HPAM decrease with the increasing gas pressure of CO_2_ and O_2_, the reasons are quite different. High-pressure CO_2_ led to a decrease in the pH of the HPAM solution, resulting in the further configuration alteration of HPAM molecules, rendering them curlier [23,24]. However, dissolved O_2_ (DO) led to oxidative degradation of HPAM and broke the polymer molecules into fragments during the long-term aging process [17,19]. These arguments are supported by the changes in the molecular weight of HPAM (Figure 12). The viscosity-average molecular weight of HPAM barely changed with the pressure of CO_2_, indicating that the reduction in viscosity and R_h_ resulted from the configuration alteration of HPAM molecules. On the contrary, the molecular weight of HPAM exhibited a drastic reduction with the increasing pressure of O_2_, showing that the degradation of HPAM was responsible for the reduction in viscosity and R_h_. Compared to CO_2_, the O_2_ in flue gas inflicted more serious damage on the long-term stability of HPAM at elevated gas pressures.

#### 2.4.2. ORP Value

**Figure 13:**
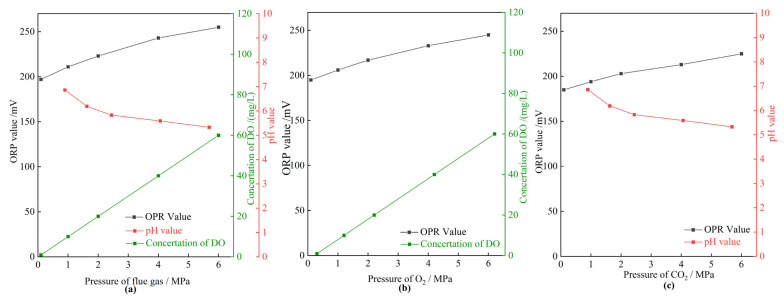
The properties of the HPAM solution in different pressures of gases. (**a**) The OPR value, concentration of DO, and pH value as a function of pressure of flue gas; (**b**) the OPR value and concentration of DO as a function of pressure of O_2_; (**c**) the OPR value and pH value as a function of pressure of CO_2_.

To obtain a deep insight into the effect mechanism of flue gas on the gel long-term stability at elevated gas pressures, the oxidation–reduction properties of the HPAM solution were characterized using the ORP value. The higher the ORP value, the stronger the oxidizing properties of a solution. The ORP value of a solution is affected by many factors, including dissolved oxygen, pH, temperature, etc. [42]. Figure 13 displays the properties of the HPAM solution, including ORP value, concentration of DO, and pH value, versus the pressure of gas. The concentration of DO in solution with different pressure of flue gas at 42 °C was calculated by Henry’s law. As shown in Figure 13a, the concentration of DO was 0.93 mg/L at atmospheric pressure. When increasing the pressure of flue gas, large quantities of O_2_ dissolved into the solution. The concentration of DO reached up to 60 mg/L in the presence of 6 MPa flue gas. Meanwhile, the pH value decreased from 6.86 to 5.33 as the pressure increased from 0.1 to 6 MPa due to the dissolution of CO_2_ into solution. Both O_2_ and CO_2_ led to an increase in ORP value (Figure 13b,c). As a result, the ORP value presented an unfavorable increase at an elevated flue gas pressure due to the comprehensive effect of O_2_ and CO_2_, indicating a stronger oxidizing property of the HPAM solution. Thus, the HPAM in the crossed-linked gel inevitably suffers from oxidizing degradation at elevated flue gas pressures.

#### 2.4.3. Long-Term Stability of Polymer Gel

**Figure 14:**
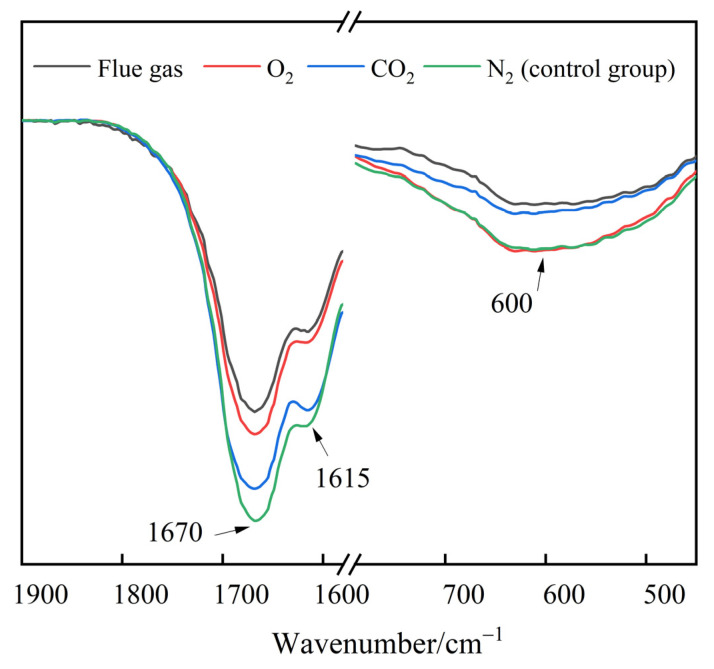
The FTIR spectra of polymer gel after being aged for 180 days.

The infrared characteristic peaks of the functional groups change with the cross-linking and degradation processes of HPAM. Therefore, the effect of flue gas on the long-term stability of polymer gels can be interpreted through FTIR analysis. The polymer gels were filled with 2 MPa of different types of gases and aged for 180 days. The FTIR spectra of polymer gels are presented in Figure 14. The broad peak at 600 cm^−1^ observed in gels was assigned to primary amine group (–NH_2_) bending vibration [43]. For the resorcinol/HMTA-based polymer gel, the –NH_2_ groups in HPAM cross-linked with the –CH_2_OH in phenolic resin, resulting in a decrease in the peak at 600 cm^−1^ and the generation of secondary amine groups (–NH–). The peak of the gels treated with flue gas and CO_2_ were much lower than those of the gels treated with N_2_ and O_2_, indicating a further cross-linking reaction caused by CO_2_. In contrast to the control group, the absorption peaks decreased significantly at 1670 cm^−1^ and 1615 cm^−1^ in flue gas and O_2_ groups, respectively. Given that the stretching peaks at 1670 cm^−1^ and 1615 cm^−1^ correspond to the C=O bond in the –CONH_2_ group and the C-O bond in the –COO^−^ group, respectively, the reduction in the absorption peaks can be attributed to the degradation of HPAM molecules and the elimination of –CONH_2_ and –COO^−^ in HPAM [44].

According to the results in Section 2.2, it is concluded that flue gas induced a stronger oxidizing property of HPAM at elevated gas pressures due to the comprehensive effect of O_2_ and CO_2_. Meanwhile, O_2_ was likely the decisive factor in the degradation of HPAM and assumed primary responsibility for the impairment of the gel’s long-term stability at elevated flue gas pressures. In addition, CO_2_ led to a greater decrease in pH at elevated gas pressures, resulting in a reduction in the R_h_ of HPAM molecules, which further impaired the long-term stability of the polymer gels.

**Figure 15:**
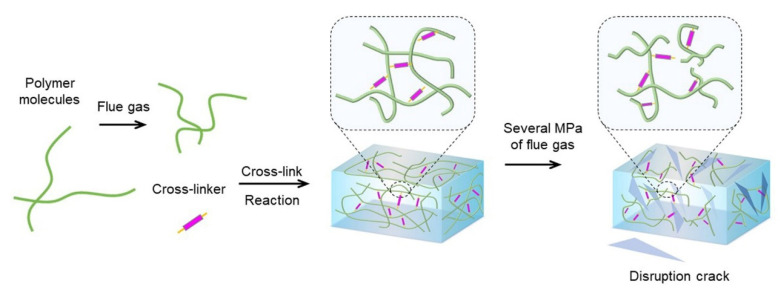
Schematic illustration of the generation and disruption of polymer gel in a flue gas environment.

Based on the above findings, the gelation process of HPAM and the disruption process of polymer gel in flue gas environment are illustrated in Figure 15. Despite the fact that the R_h_ of the HPAM molecules decreased with the dissolution of CO_2_, the flue gas expedited the generation of cross-linkers, and then promoted the cross-linking process with HPAM, consequently leading to a shorter gelation time and enhanced gel strength. After forming into a bulk gel, several MPa of flue gas was injected into the reservoir for dozens of days. A further cross-linking reaction occurred due to the CO_2_. However, the composited O_2_ induced the degradation of HPAM and disrupted the cross-linked networks of the polymer gel, threatening the gel’s long-term stability. To address the adverse effects caused by flue gas, it is highly desirable to advance the polymer gel formulation by adding oxygen scavengers or strengthening additives.

## 3. Conclusions

The gelation behavior and gel performance of resorcinol/ HMTA-based polymer gels were explored with flue gas treatment. Flue gas inflicted a complicated effect on the gelation behavior and the gel performance. The results revealed the following:

1. The flue gas facilitated the gelation performance of resorcinol/HMTA gel, which was beneficial to the in situ polymer gel treatment. However, it threatened the gel long-term stability, especially in high-pressure conditions;
2. Owing to the composited CO_2_, the flue gas altered the configuration of HPAM, resulting in a rapid reduction in the R_h_ and initial viscosity of HPAM solution. However, CO_2_ expedited the decomposition rate of HMTA into formaldehyde, and, thus, promoted the cross-linking process, leading to a shorter gelation time and an enhanced gel strength;
3. Flue gas increased the ORP value and induced a stronger oxidizing property of HPAM at elevated flue gas pressures due to the comprehensive effect of O_2_ and CO_2_;
4. The O_2_ in flue gas played a leading role in the degradation of HPAM and assumed primary responsibility for the impairment of the gel long-term stability at elevated flue gas pressures. To address the adverse effects caused by flue gas and to meet the effective mobility control requirements in flue gas flooding reservoirs, it is highly desirable to develop polymer gels by adding oxygen scavengers or strengthening additives.

## 4. Materials and Methods

### 4.1. Materials

Low molecular weight (2 million) HPAM with a hydrolysis degree of 4% was supplied by SNF (China) Flocculant, Co., Ltd. Resorcinol (99.0%, purity), HMTA (99.0%, purity), and acetic acid (99.5%, purity) were purchased from Sigma-Aldrich and used as cross-linkers. Four types of gases, including flue gas (80%N_2_, 17% CO_2_, and 3% O_2_), composited CO_2_ (83% N_2_ and 17% CO_2_), composited O_2_ (97% N_2_ and 3% O_2_), and N_2_, were obtained from Shenkai Gas Co. Ltd., Shanghai, China. A formation brine obtained from the Hong oilfield was used to prepare gelant samples, and its composition is shown in Table 1.

### 4.2. Methods

#### 4.2.1. Preparation of Polymer Gel

**Figure 16:**
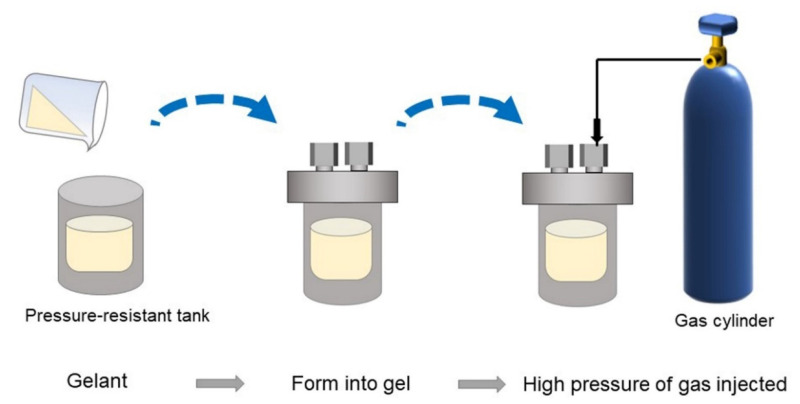
Experimental flow for investigating long-term stability of gels at elevated gas pressures.

The gelants were obtained by evenly mixing HPAM stock solution with cross-linker solution, including resorcinol, HMTA, and acetic acid, in different proportions. Subsequently, the gelant samples were saturated with flue gas, N_2_, CO_2_, and O_2_ separately and sealed in glass bottles. Then, they were placed in a 42 °C water bath for further study. Furthermore, the long-term stabilities of gels at elevated gas pressures were also assessed. The experimental flow is depicted in Figure 16. As shown, firstly, the gelants were prepared, transferred to a pressure-resistant tank, and subsequently placed at 42 °C for a sufficient time. After bulk gel was formed, the tank was injected with different pressures of gases and set to 42 °C again for 180 days for further study.

#### 4.2.2. Gel Rheological Properties

The storage modulus (G′) of polymer gels was measured on an Anton Paar MCR92 rheometer to quantify the gel strength. The samples were filled into the space between the PP25 rotor and the base plate. Furthermore, a strain sweep was conducted within the range of 1–500% while keeping the frequency at 1 Hz. The G′ derived from the linear viscoelastic region was adopted as the criterion of gel strength. In addition, the gelation time corresponded to the time when the G′ sharply increased in the aging process. The rheological variation of HPAM solutions was measured using a Brookfield viscometer with a shear rate of 7.34 s^−1^.

#### 4.2.3. DLS Measurements

The R_h_ of the HPAM solutions was adopted to examine the variation of the polymer configuration. First of all, the HPAM solutions were diluted to 5 mg/L and filtered through a membrane filter to eliminate the large suspended particles. Then, the diluted HPAM solutions were saturated with different pressures of flue gas and aged for sufficient time at 42 °C. The R_h_ of the HPAM solutions was analyzed.

#### 4.2.4. Formaldehyde Concentration Measurements

The HMTA solution tends to decompose into formaldehyde in acidic conditions at 42 °C. The concentration of formaldehyde can be determined using a pH meter. Firstly, 50 mL of HMTA–acetic acid solution was obtained by mixing acetic acid and the HMTA solution together. The mass fractions of acetic acid and HMTA were 0.2 wt% and 0.3 wt%, respectively. Then, the mixed solution was aged for 48 h at 42 °C, and the pH value was adjusted to 3.5 with 1 mol/L HCl. Afterwards, 25 mL of 10 wt% hydroxylamine hydrochloride was added to the mixed solution. Stirring was carried out for 10 min to induce a complete oximation reaction. The HCl was generated during the reaction process, and the pH decreased to a certain value. Finally, 1 mol/L sodium hydroxide was titrated to recover the pH value to 3.5 again. The concentration of formaldehyde was calculated by the following equation [45]:

*w* = 3*c*(*V*_1_ − *V*_0_)/50 × 100% (1)

where *w* is the formaldehyde concentration, *c* is the NaOH concentration, *V*_1_ is the volume of NaOH used in samples, and *V*_0_ is the volume of NaOH used in the blank sample.

#### 4.2.5. FTIR Measurements

Here, FTIR spectra analyses were adopted to characterize the composition and transformation of functional groups in polymer gels. The polymer gels were treated with 2 MPa of different types of gases and aged for 180 days. Then, gel samples were dehydrated in a vacuum drying oven. The FTIR specimens were prepared by the potassium bromide (KBr) pellet method. The proportion of gel sample to KBr in all pellets was identical (1:100) to ensure data consistency. The spectral analyses were performed on an FTIR spectrometer within the wave number range of 4000 cm^−1^ to 400 cm^−1^.

## Figures and Tables

**Table 1:** Ion composition of the formation brine.

Ion Type	K^+^ + Na^+^	Ca^2+^	Mg^2+^	Cl^−^	HCO_3_^−^	SO_4_^2−^	Total Salinity
Concentration /(mg/L)	1897.63	64.92	5.45	2525	850.12	49.7	5392.82

## Data Availability

Not applicable.
